# Three-dimensional kinematic evaluation of lateral suture stabilization in an *in vitro* canine cranial cruciate deficient stifle model

**DOI:** 10.1371/journal.pone.0261187

**Published:** 2021-12-20

**Authors:** Laura-Isabela Del Carpio, Yvan Petit, Lucien Diotalevi, Elisabeth Laroche, Annie Levasseur, Bertrand Lussier

**Affiliations:** 1 Department of Clinical Sciences, Faculty of Veterinary Medicine, University of Montreal, Saint-Hyacinthe, Quebec, Canada; 2 Department of Mechanical Engineering, École de Technologie Supérieure, Montreal, Quebec, Canada; 3 CIUSSS-NÎM Research Center, Sacré-Cœur Hospital of Montreal, Montreal, Quebec, Canada; 4 GREPAQ (Research Group in Animal Pharmacology of Quebec), Faculty of Veterinary Medicine, University of Montreal, Saint-Hyacinthe, Quebec, Canada; 5 Osteoarthritis Research Unit, Hospital Research Centre (CRCHUM), Université de Montréal, Montreal, Quebec, Canada; University of Memphis, UNITED STATES

## Abstract

The impact of surgical correction of cranial cruciate ligament rupture (CCLR) on 3D kinematics has not been thoroughly evaluated in dogs. The success of current techniques remains limited, as illustrated by suboptimal weightbearing and progression of osteoarthritis. The inability to restore the stifle’s 3D kinematics might be a key element in understanding these suboptimal outcomes. The objective of this study was to evaluate the impact of lateral suture stabilization (LSS) on the 3D kinematics of the canine stifle joint. We hypothesized that LSS would not restore 3D kinematics in our model. Ten cadaveric pelvic limbs collected from large dogs (25–40 kg) were tested using a previously validated apparatus that simulates gait. Three experimental conditions were compared: (a) intact stifle; (b) unstable stifle following cranial cruciate ligament transection (CCLt) and (c) CCLt stabilized by LSS. Three-dimensional kinematics were collected through 5 loading cycles simulating the stance phase of gait and curves were analyzed using a Wilcoxon signed-rank test. LSS restored baseline kinematics for the entire stance phase for cranial and lateromedial translation, flexion, and abduction. It restored distraction over 90% of the stance phase. Internal rotation was limited, but not restored. This in vitro study had limitations, as it used a simplified model of stifle motion and weight-bearing. The results of this study report that LSS can restore physiologic 3D kinematics largely comparable to those of healthy stifles. Suboptimal outcome in patients following CCLR stabilization by LSS may therefore result from causes other than immediate postoperative abnormal 3D kinematics.

## Introduction

Cranial cruciate ligament rupture (CCLR) is the most common orthopedic condition affecting dogs [1]. It modifies joint kinematics [2] and contact mechanics [3], therefore creating joint instability. While various surgical techniques aim to restore normal limb function following CCLR, the optimal treatment for this condition remains an ongoing debate. The success of current techniques remains limited as they are not able to fully restore weightbearing [4–6] or slow the development of osteoarthritis [5]. Restoring joint stability without aiming to restore joint kinematics might be a key element in understanding the suboptimal success outcome of surgical CCLR correction.

The impact of surgical CCLR stabilization on the joint’s 3D kinematics has not been thoroughly evaluated in the canine stifle. One of the most frequently used techniques [7, 8], lateral suture stabilization (LSS), provides mixed success outcomes. While this technique is reported to be inferior to others [4, 9], a recent experimental study [10] has shown that normal weightbearing can be obtained with LSS one year after cranial cruciate ligament transection. Other studies have also reported that LSS is non-inferior to TPLO when comparing objective outcomes in clinically afflicted dogs [5, 6, 11]. The ability of LSS to restore joint kinematics has not been thoroughly evaluated, and the lack of understanding of the impacts of the technique on joint kinematics could explain these mixed results. *In vitro* models are widely used for 3D kinematics evaluation because they limit variation and are more ethically acceptable. In this study, we used a validated testing jig that simulates canine gait in isotonic conditions, taking into account weight-bearing, quadriceps muscle force and whole limb motion [12] to evaluate LSS. The objective of this study was to evaluate the impact of LSS on the 3D kinematics of the canine stifle joint. We hypothesized that LSS would not restore normal 3D kinematics in our model.

## Materials and methods

### Sample size

Sample size was determined based on calculations from a previous study [16] using the same jig and methodology for a power of 90% and an alpha of 0.05. It was calculated for the anticipated mean values of the two most clinically relevant parameters: cranial translation and internal rotation. Based on these statistical parameters, minimal sample size required was n = 4 in each group (4 stable stifles and 4 unstable stifles). We opted to use 10 stifles to take into account technical problems which could lead to group attrition.

### Specimen selection

Ten pelvic limbs (n = 10) were collected from 7 mature dogs euthanized for reasons unrelated to this study. The cadavers were provided by donation through the University. All dogs were previously euthanized for reasons not related to the study. We recuperated the limbs after other investigators harvested sample organs for research and/or teaching purposes in accordance with the 3R rules by Russell and Burch. No approval from our IACUC was required.

The dogs weighed from 25 to 40 kg and were of various large breeds. Macroscopic examination and palpation of the stifles was performed by a surgeon (ACVS) to rule out any gross abnormalities (malformation, instability, crepitus). Orthogonal radiographs of the limbs were taken to detect evidence of stifle pathology and to exclude stifles with excessive tibial plateau angle (>35°) [13]. Based on these criteria, three stifles with abnormalities were excluded from the study. Selected limbs were frozen at -20°C until specimen preparation.

### Specimen preparation

Specimens were prepared as previously described for the testing apparatus [12]. Before preparation, specimens were thawed at 4°C for 48 hours. For instrumentation, the tarsus was disarticulated and the limbs were dissected to remove skin, preserving the muscles and the structures surrounding the stifle joint. Rigid bodies were fixed to the tibia and femur using partially threaded fixation pins. Optoelectronic diodes were installed on these rigid bodies during testing, allowing kinematic data acquisition. The entire pelvic limb, including the two rigid bodies, was scanned (Skyscan 1176, Bruker BioSpin Corp., Ma, USA; 30mA, 120kv, slice thickness 0.625mm, interval 0.625). All limbs were virtually reconstructed (3D-Slicer v.4.10.2, http://www.slicer.org), and anatomical landmarks were used to define a joint coordinate system [12]. After the scan, the distal tibia and proximal femur were sectioned, and the limbs were cemented using polyester resin (Bondo Body Filler, Bondo Corporation, Atlanta, GA) into PVC pipes using a custom-made centering jig to match the artificial articulations of the tarsus and hip in the testing apparatus.

### Testing apparatus

**Fig 1:**
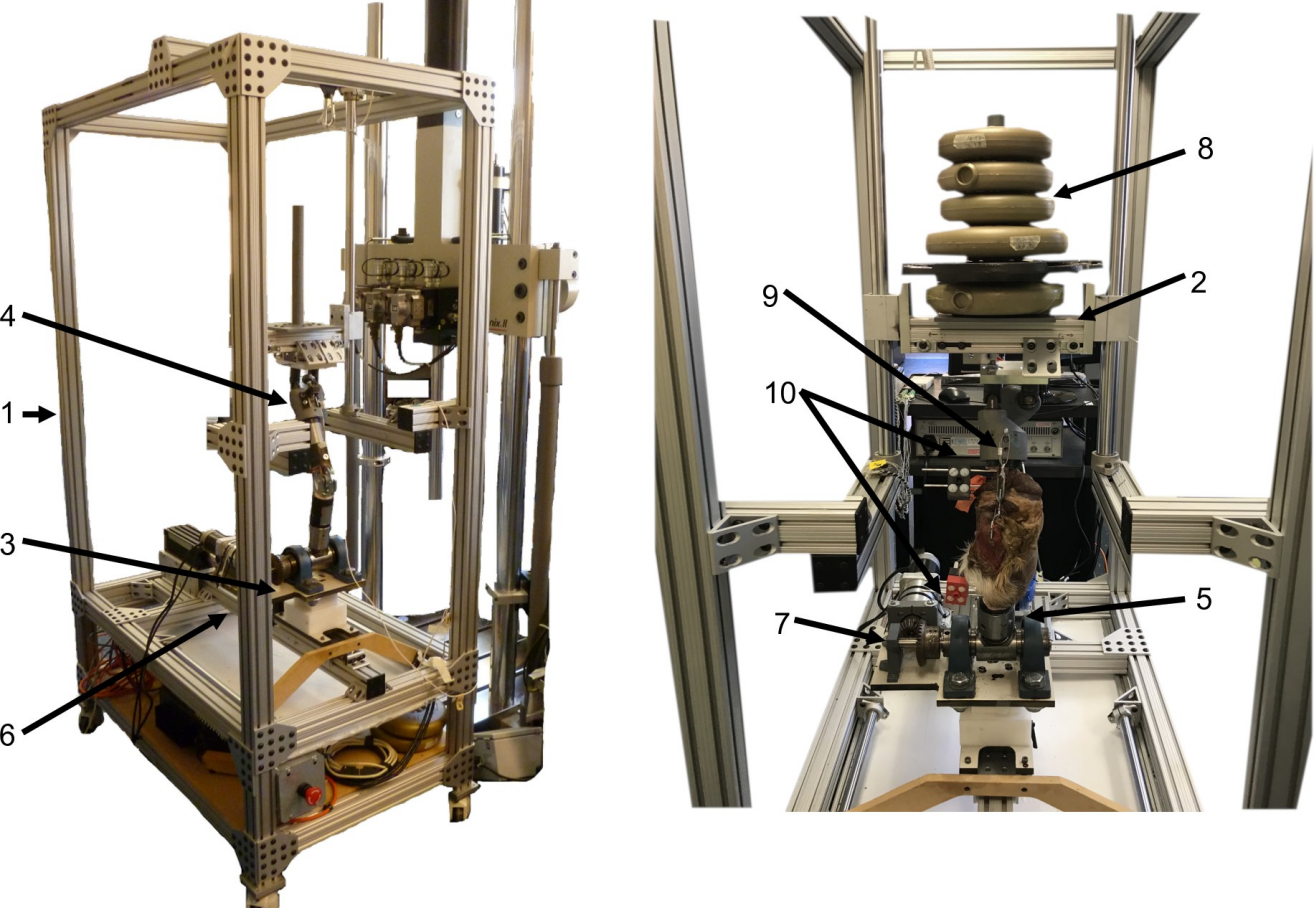
The testing apparatus used for the study with a loaded limb in place. Cranio-caudal views (A) Oblique. (B) Frontal; 1. Frame; 2. Upper platform; 3. Lower platform; 4. Artificial hip joint; 5. Artificial tarsal joint; 6. Linear actuator; 7. Rotating actuator; 8. Weights, centralized over hip joint; 9. Turnbuckle attached to proximal tibia and patella; 10. Rigid bodies fixated in femur (black) and tibia (red).

The apparatus used for this study simulates the stance phase of gait for the canine hindlimb and has been validated for kinematic evaluation of the normal and cranial cruciate-deficient stifles [12] (Fig 1). For testing, the limb was mounted in the artificial hip and tarsus articulations and motion was created by two actuators, one linear and one rotational, programmed to simulate the stance phase of trot. The apparatus operated in quasi-dynamic mode, as the two actuators positioned the limb at each 10% of stance and paused for 1 second at this position while kinematic data was recorded.

### Data acquisition

3D kinematics were recorded using an optoelectronic system (Optotrak 3020, NDI, Waterloo, Canada). Five optoelectronic diodes were placed on each rigid body, and their relative position was captured during gait. The six degrees of freedom for the stifle joint (flexion/extension; abduction/adduction; internal/external rotation of the tibia; cranio/caudal, medio/lateral, and proximo/distal translations) were computed at each 10% interval of the stance phase using Euler angles with the method of Grood and Suntay [14]. The values were then interpolated using a robust quadratic algorithm to generate continuous curves across the entire stance phase.

### Testing protocol

To simulate weight-bearing, 45% of body weight (BW) was applied on the upper platform of the testing apparatus. This value was the highest weight-bearing that the apparatus allowed without inducing significant vibrations influencing data collection. Tension in the quadriceps was simulated using a turnbuckle applying adequate tension from the artificial hip articulation to the patella as previously described [12].

Data was collected over 5 gait cycles for each one of the 3 experimental conditions: intact stifle, unstable stifle following cranial cruciate ligament transection (CCLt), and stabilized stifle by LSS. All limbs underwent preconditioning for 10 cycles as previously defined [12]. First, data was collected for the intact stifle. Then the limb was disassembled from the artificial hip articulation and the cranial cruciate ligament was transected through a medial arthrotomy which was then sutured. The limb was reassembled in the hip articulation, setting it back to its original position by reapplying the same number of turns in tightening the turnbuckle simulating quadriceps tension and resetting the joint angles to the starting position. Second, data was collected for the unstable stifle. Then with the limb still mounted in the apparatus, LSS was performed using two strands of 80 lb nylon leader line (Securos, Fiskdale, MA) by a single boarded surgeon. The suture was passed around the lateral fabella from caudo-proximal to cranio-distal, then through a hole previously drilled in the tibial tuberosity and under the patellar tendon. The first strand was tensioned on the lateral aspect of the stifle to neutralize cranial drawer and secured using crimp clamps. The stifle underwent 20 cycles to assess any loosening in the suture as described by Choate *et al*. [15]. Suture loosening was assessed by cranial drawer test. If there was no residual cranial drawer after 20 cycles, the second strand was tensioned as tight as the first using crimp clamps; it was tensioned until we observed that the first strand was starting to loosen/buckle, as it is done clinically when performing LSS. If there was evidence of loosening of the first strand after the 20 cycles, the second strand was tightened to neutralize any residual cranial drawer and 20 cycles were repeated. No limbs were observed to have residual cranial drawer after the tightening of the second strand. If there had been residual cranial drawer after the two strands were tightened, we would have cut one of the strands and replaced a new one using adequate tension. The protocol would then have followed as described with 20 additional cycles. Finally, data was collected for the stabilized stifle.

### Data analysis

Kinematic curves were analyzed to evaluate the impact of LSS on joint kinematics. For each experimental condition (intact stifle, CCLt stifle, LSS stifle), the curves were computed to represent the average of all gait cycles for the 10 specimens. The curves were first qualitatively analyzed (amplitude, shape, deviation from baseline). A statistical analysis was then conducted to assess return to baseline using a Wilcoxon signed-rank test with significance set at *p* < 0.05. Kinematic curves were compared for the 3 experimental conditions over the whole stance phase for the 6 degrees of freedom of the stifle.

## Results

**Fig 2:**
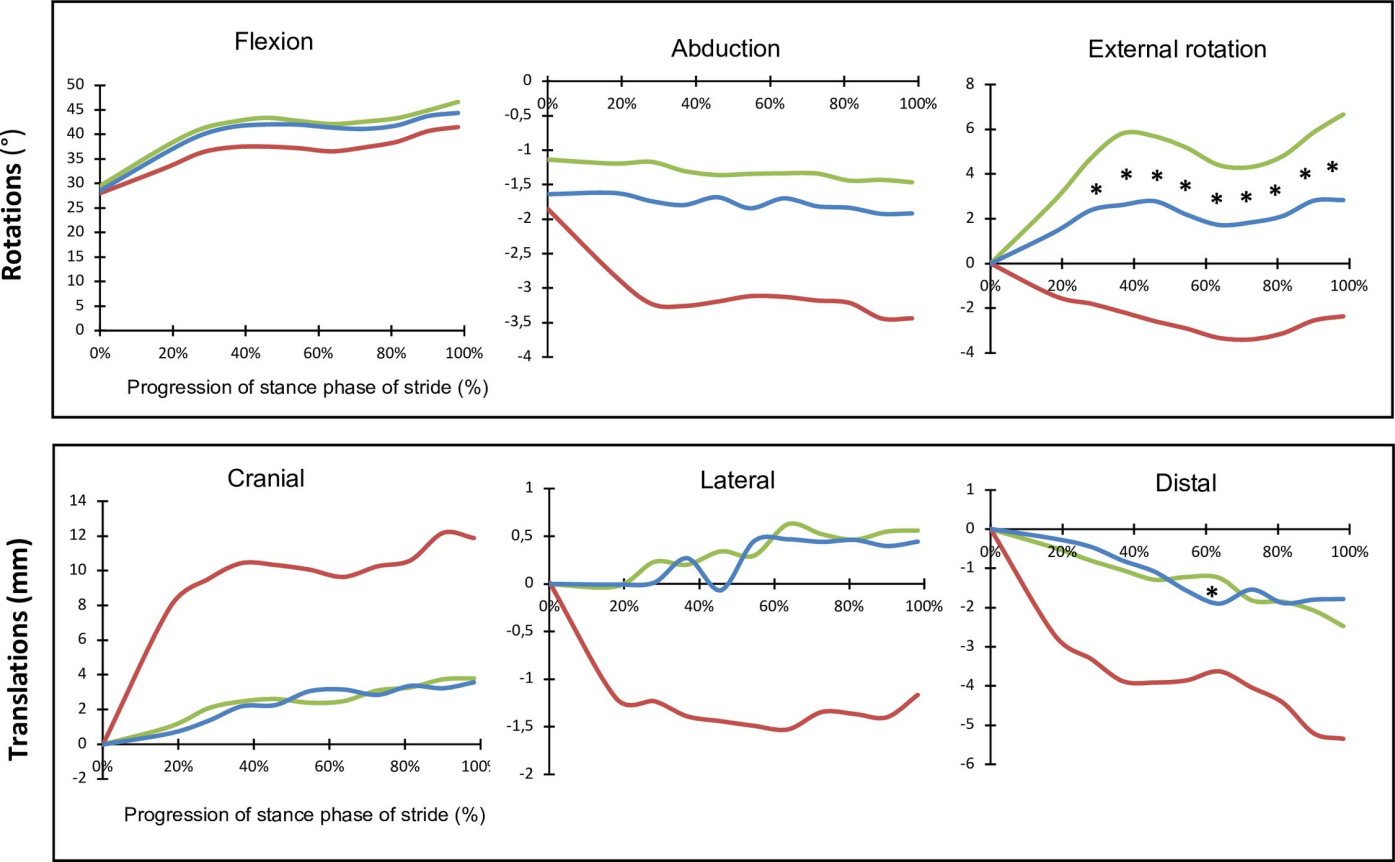
Three-dimensional kinematics for intact (green), cranial cruciate ligament deficient (red) and lateral suture stabilized (blue) stifles through the stance phase of stride. Curves represent the mean values for 10 stifles through 5 cycles each. * Statistically significant difference (using Wilcoxon signed-rank test) at that time point between intact and stabilized stifles.

Data is summarized in Fig 2. The curves represent the mean values for all the specimens tested for 5 loading cycles each. They represent the 3 translations and 3 rotations of the tibia in relation to the femur over the progression of the stance phase, with statistical comparisons between intact and stabilized stifles.

Cranial cruciate ligament transection resulted in cranial, proximal and medial translation, as well as extension, adduction, and internal rotation of the tibia in relation to the femur. With LSS stabilization of the stifle, kinematics were restored to baseline for the entire stance phase for cranial and lateral translations, for flexion, and for abduction. Distraction was restored to baseline over 90% of the stance phase. Between 60% to 70% of the stance phase, there was a significant difference between intact and LSS stifles, with LSS causing 0.6 mm of compression compared to the intact stifle (*p* < 0.05). External rotation was restored for the first 40% of the stance phase. For the rest of the stance phase, LSS limited the internal rotation resulting from CCLt, but the stabilized stifle remained significantly less externally rotated than the intact stifle (*p* < 0.05). Moreover, LSS did not significantly influence the range of motion in the sagittal plane (*p* < 0.05).

## Discussion

Using a validated testing apparatus that simulates the weight-bearing stance phase of the canine gait, this study reported 3D kinematic curves in three experimental conditions: intact, CCL-deficient and LSS stifles, for the 6 degrees of freedom of the stifle joint. As reported in the study validating the apparatus [12] and in a previous study performed with this testing jig [16], the changes induced by CCLt in our study were comparable to the *in vivo* kinematic data for CCLR in the literature [2].

### Translations

#### Cranio-caudal translation

LSS created a cranio-caudal kinematic curve similar in shape to the intact stifle, with no statistical difference between the two groups at any point during stance. This suggests that LSS was effective in neutralizing cranial tibial translation in our model. This has also been reported in previous biomechanical studies [17, 18] and at three different moments of the stance phase [19].

#### Medio-lateral translation

LSS neutralized the medial translation in our CCLt model and restored baseline kinematics throughout stance phase for this motion. To our knowledge, medio-lateral translation following LSS has only been evaluated by one other study [19]. This study compared kinematics for two tibial bone tunnel sites, the tibial tuberosity and the extensor groove, and found that LSS using both sites resulted in significant lateral translation. The discrepancy between our results and these could be explained by variation in suture tensioning as the anchor points of LSS pull on the tibia laterally and caudally [19] with different forces [6] depending on the tension applied to the suture. With adequate suture tensioning that successfully neutralized cranial drawer, this phenomenon was not observed in our study as latero-medial translation was similar to the intact stifle throughout the stance phase, emphasizing that suture tension might have an impact on the 3D kinematics of the stifle joint. It has been reported that over tightening of an extra-articular prothesis also results in compression of the lateral femoro-tibial compartment [20]. Overtension might be a key element in the development of osteoarthritis associated with LSS.

#### Proximo-distal translation

With stabilization of the stifle by LSS, proximo-distal kinematics returned to baseline for 90% of the stance phase, except from 60% to 70%. In this part of the stance phase, LSS caused statistically significant compression compared to the intact stifle (p < 0.05). As seen graphically in Fig 1, the difference between the mean values for LSS and intact stifles was less than 0.5 mm of translation at that point of the stance phase. To our knowledge, only one study evaluated this motion for LSS [19] and found that proximo-distal translation was returned to baseline for early-, mid- and late-stance. The previous study used weight-bearing of 30% and simulated the stance phase of walk, which could additionally explain these results differing from what was found in the present study. The significant difference between the LSS and normal stifles at that point of the stance phase could be explained by the small sample size (n = 10) and by one limb that had a significantly larger compression following LSS (2.63 mm of proximal tibial translation versus a mean of 0.70 mm for the other limbs). Despite this 10% where kinematics were statistically different between the two groups, LSS resulted in physiologic kinematics for this motion. These results must consider that only 45% BW was used in our model. We cannot extrapolate on the degree of compression *in vivo* when 72% BW is applied to the stifle joint at trot.

### Rotations

#### Flexion/Extension

In our study, LSS stabilization resulted in kinematics that were restored to baseline for flexion. To our knowledge, this motion was only evaluated in one other study [11]. This kinematic study found that dogs at a mean of 8 years post-LSS stabilization had stifle flexion similar to clinically normal dogs. Our study reported that LSS seems to be effective at restoring flexion immediately after stabilization during the stance phase of trot.

#### Abduction/Adduction

With LSS, kinematics were restored to baseline for abduction and adduction motions in our model. This has been reported in live dogs at a mean of 8 years post-LSS stabilization [11]. However, some *in vitro* studies have reported LSS to cause significant abduction of the stifle [19, 21]. As mentioned previously, the amount of tension applied to the lateral prosthesis could be responsible for increased abduction reported in these studies, as the tibial tuberosity is pulled outwards in the direction of the lateral fabella with increased tension. Further studies are required to better understand the effect of suture tensioning on 3D kinematics in extracapsular stabilization techniques.

#### Internal/External rotation

With LSS, the internal rotation caused by CCLt was neutralized to baseline for the first 40% of stance but was only limited for the rest of the stance phase. The tibia was brought back to a physiologic external rotation that was statistically inferior to the intact stifle (*p* < 0.05). Other studies that evaluated this motion found different results. Headrick *et al*. reported kinematics similar to normal for this motion in dogs that had been operated 8 years before data collection [11]. In an *in vitro* non-weightbearing model, Chailleux *et al*. found that a similar technique, the modified retinacular imbrication technique with medial and lateral prostheses, caused significant external rotation of the tibia and raised concerns about the impact of this on compression of the lateral compartment of the stifle joint [21]. As mentioned previously, it has been reported that increasing suture tension causes amplification of contact pressures in the lateral compartment of the stifle [20]. In a different weight-bearing *in vitro* study, Aulakh *et al*. observed similar excessive external rotation and hypothesized that the suture anchor in the tibial tuberosity acted as a lever to pull the tibia caudally and laterally, resulting in external rotation [19]. Excessive external rotation was also reported after LSS by Snow *et al*. in an *in vitro* model under torsional loading [17]. With the suture tensioning technique used in our protocol, the LSS procedure did not cause this excessive external rotation and successfully returned the stifle to physiologic kinematics for this motion without fully restoring baseline. It is interesting to note that this is in contrast to what has been recently reported with tibial plateau leveling osteotomy (TPLO) where dogs with persistent cranial tibial subluxation have been reported to also have internal tibial rotation [22], which has led to combine TPLO with an extracapsular technique to address this issue [23].

Our hypothesis was rejected as LSS restored 3D kinematics largely comparable to those of intact stifles. These results suggest that with adequate suture tensioning, LSS can lead to physiologic kinematics. It is our belief that LSS tensioning should aim to apply a tension that is only sufficient to limit cranial drawer but not excessive in order to limit its impact on 3D kinematics in the other planes. Variability associated with LSS tensioning has been reported within and between surgeons and is suggested to lead to inconsistent clinical outcomes [24]. Further studies are needed to assess the impact of excessive LSS suture tensioning on 3D kinematics in order to provide clearer clinical guidelines.

These results raise an interesting concern as to why patients stabilized by LSS, whose kinematics can be returned to physiologic motions, are still affected with the progression of osteoarthritis? In experimental studies where the CCL was transected and the stifle was quickly stabilized by LSS, dogs were reported to be brought back to normal weight-bearing at 20 weeks [25], one year [10], and 8 years [11] following stabilization. In a healthy stifle with CCL experimental transection, LSS is therefore effective at restoring normal weight-bearing and, as the present study has shown, also seems to be effective at restoring normal kinematics. What can explain the suboptimal outcomes that we see in naturally occurring CCLR where weight-bearing is inferior to normal [4–6] and osteoarthritis progresses [5, 26, 27] despite stabilization?

Dogs with naturally occurring CCLR and subsequent osteoarthritis have been reported to have increased sensory sensitivity in the affected limb and at remote sites [28–31], suggesting they are likely affected by central sensitization. These newly elucidated aspects of CCLR in dogs should require additional case management considerations in addition to surgical stabilization that is solely focused on joint stabilization. In human medicine, a new anterior cruciate ligament (ACL) repair technique that is able to bridge the gap between the torn ends of the ACL and promote its healing [32] has been reported to successfully minimize post-traumatic osteoarthritis [33]. These findings suggest that preserving the native ACL, particularly the proprioceptive fibers, could improve proprioceptive function after surgery and limit post-traumatic osteoarthritis [32]. Further studies are needed in canine patients to find ways to prevent the impacts of degenerative joint disease beyond mechanical stabilization.

As all in vitro studies, this study had limitations. First, all muscle forces involved in stifle stabilization could not be replicated. Only the quadriceps tension was simulated, and this tension was applied using a simplified model of physiological tension as it was linear. Second, weight-bearing remains a simplification of reality in this model. Weight-bearing had to be limited to 45% of body weight and had to remain constant throughout gait and was therefore inferior to what is normally found at trot speed [34]. Weight-bearing had to be limited because of excessive vibrations in the apparatus influencing data collection. Third, our model used quasi-dynamic motion in order to decrease vibrations in the apparatus, requesting data to be interpolated in order to create continuous kinematic curves. Fourth, the sample size that was used was small. Finally, our *in vitro* model only assessed the impacts of LSS on kinematics immediately after stabilization.

## Conclusions

Using a validated testing apparatus that simulates the weight-bearing stance phase of canine gait, the present study reported that LSS restored physiological 3D kinematics following CCL transection. LSS resulted in successful cranio-caudal stabilization and did not cause excessive external rotation or abduction. Combined with results from previous experimental studies where weight-bearing was restored by LSS [10, 11, 25], the results of the present study suggest that the suboptimal outcomes reported in patients stabilized by LSS might not be caused by an ineffective technique, but rather by other aspects of degenerative joint disease that are not currently taken into account in case management.

## Supporting information

S1 Data (XLSX)

## References

[1] Johnson J, Austin C, Breur GJ. Incidence of canine appendicular musculoskeletal disorders in 16 veterinary teaching hospitals from 1980 through 1989. Vet Comp Orthop Traumatol. 1994;7:56–69.

[2] Korvick DL, Pijanowski GJ, Schaeffer DJ. Three-dimensional kinematics of the intact and cranial cruciate ligament-deficient stifle of dogs. J Biomech. 1994;27(1):77–87. doi: 10.1016/0021-9290(94)90034-5 8106538

[3] Kim SE, Pozzi A, Banks SA, Conrad BP, Lewis DD. Effect of cranial cruciate ligament deficiency, tibial plateau leveling osteotomy, and tibial tuberosity advancement on contact mechanics and alignment of the stifle in flexion. Vet Surg. 2010;39(3):363–70. doi: 10.1111/j.1532-950X.2010.00655.x 20522216

[4] Gordon-Evans WJ, Griffon DJ, Bubb C, Knap KM, Sullivan M, Evans RB. Comparison of lateral fabellar suture and tibial plateau leveling osteotomy techniques for treatment of dogs with cranial cruciate ligament disease. J Am Vet Med Assoc. 2013;243(5):675–80. doi: 10.2460/javma.243.5.675 23971847

[5] Au KK, Gordon-Evans WJ, Dunning D, O’Dell-Anderson KJ, Knap KE, Griffon D, et al. Comparison of short- and long-term function and radiographic osteoarthrosis in dogs after postoperative physical rehabilitation and tibial plateau leveling osteotomy or lateral fabellar suture stabilization. Vet Surg. 2010;39(2):173–80. doi: 10.1111/j.1532-950X.2009.00628.x 20210964

[6] Conzemius MG, Evans RB, Besancon MF, Gordon WJ, Horstman CL, Hoefle WD, et al. Effect of surgical technique on limb function after surgery for rupture of the cranial cruciate ligament in dogs. J Am Vet Med Assoc. 2005;226(2):232–6. doi: 10.2460/javma.2005.226.232 15706973

[7] Comerford E, Forster K, Gorton K, Maddox T. Management of cranial cruciate ligament rupture in small dogs: a questionnaire study. Vet Comp Orthop Traumatol. 2013;26(6):493–7. doi: 10.3415/VCOT-12-06-0070 24008348

[8] Duerr FM, Martin KW, Rishniw M, Palmer RH, Selmic LE. Treatment of canine cranial cruciate ligament disease. A survey of ACVS Diplomates and primary care veterinarians. Vet Comp Orthop Traumatol. 2014;27(6):478–83. doi: 10.3415/VCOT-14-03-0047 25328024

[9] Nelson SA, Krotscheck U, Rawlinson J, Todhunter RJ, Zhang Z, Mohammed H. Long-term functional outcome of tibial plateau leveling osteotomy versus extracapsular repair in a heterogeneous population of dogs. Vet Surg. 2013;42(1):38–50. doi: 10.1111/j.1532-950X.2012.01052.x 23153073

[10] Lussier B, Gagnon A, Moreau M, Pelletier JP, Troncy E. Peak vertical force in a stabilized canine cranial cruciate deficient stifle model: A one-year follow-up. Can J Vet Res. 2018;82(2):159–61. 29755197 PMC5914080

[11] Headrick JF, Zhang S, Millard RP, Rohrbach BW, Weigel JP, Millis DL. Use of an inverse dynamics method to compare the three-dimensional motion of the pelvic limb among clinically normal dogs and dogs with cranial cruciate ligament-deficient stifle joints following tibial plateau leveling osteotomy or lateral fabellar-tibial suture stabilization. Am J Vet Res. 2014;75(6):554–64. doi: 10.2460/ajvr.75.6.554 24866511

[12] Hagemeister N, Lussier B, Jaafar E, Clement J, Petit Y. Validation of an experimental testing apparatus simulating the stance phase of a canine pelvic limb at trot in the normal and the cranial cruciate-deficient stifle: an in vitro kinematic study. Vet Surg. 2010;39(3):390–7. doi: 10.1111/j.1532-950X.2010.00673.x 20522219

[13] Duerr FM, Duncan CG, Savicky RS, Park RD, Egger EL, Palmer RH. Comparison of surgical treatment options for cranial cruciate ligament disease in large-breed dogs with excessive tibial plateau angle. Vet Surg. 2008;37(1):49–62. doi: 10.1111/j.1532-950X.2007.00348.x 18199057

[14] Grood ES, Suntay WJ. A joint coordinate system for the clinical description of three-dimensional motions: application to the knee. J Biomech Eng. 1983;105(2):136–44. doi: 10.1115/1.3138397 6865355

[15] Choate CJ, Lewis DD, Conrad BP, Horodyski MB, Pozzi A. Assessment of the craniocaudal stability of four extracapsular stabilization techniques during two cyclic loading protocols: a cadaver study. Vet Surg. 2013;42(7):853–9. doi: 10.1111/j.1532-950X.2013.12049.x 24033745

[16] Laugier M, Tremblay J, Petit Y, Grignon-Lemieux A, Levasseur A, Lussier B. Three-dimensional kinematic evaluation of Tightrope CCL in a canine in vitro cranial cruciate deficient stifle model. Can J Vet Res. 2019;83(4):317–21. 31571733 PMC6753885

[17] Snow LA, White R, Gustafson S, Xie L, Hosgood G, Monroe WT, et al. Ex vivo comparison of three surgical techniques to stabilize canine cranial cruciate ligament deficient stifles. Vet Surg. 2010;39(2):195–207. doi: 10.1111/j.1532-950X.2009.00635.x 20210967

[18] Harper TA, Martin RA, Ward DL, Grant JW. An in vitro study to determine the effectiveness of a patellar ligament/fascia lata graft and new tibial suture anchor points for extracapsular stabilization of the cranial cruciate ligament-deficient stifle in the dog. Vet Surg. 2004;33(5):531–41. doi: 10.1111/j.1532-950X.2004.04075.x 15362993

[19] Aulakh KS, Harper TA, Lanz OI, D’Amico LL, Butler JR, McLaughlin RM, et al. Effect of tibial insertion site for lateral suture stabilization on the kinematics of the cranial cruciate ligament deficient-stifle during early, middle and late stance: an in vitro study. Vet Comp Orthop Traumatol. 2013;26(3):208–17. doi: 10.3415/VCOT-12-04-0051 23460337

[20] Tonks CA, Pozzi A, Ling HY, Lewis DD. The effects of extra-articular suture tension on contact mechanics of the lateral compartment of cadaveric stifles treated with the TightRope CCL or lateral suture technique. Vet Surg. 2010;39(3):343–9. doi: 10.1111/j.1532-950X.2010.00664.x 20522214

[21] Chailleux N, Lussier B, De Guise J, Chevalier Y, Hagemeister N. In vitro 3-dimensional kinematic evaluation of 2 corrective operations for cranial cruciate ligament-deficient stifle. Can J Vet Res. 2007;71(3):175–80. 17695591 PMC1899862

[22] Tinga S, Kim SE, Banks SA, Jones SC, Park BH, Burtch M, et al. Femorotibial kinematics in dogs treated with tibial plateau leveling osteotomy for cranial cruciate ligament insufficiency: An in vivo fluoroscopic analysis during walking. Vet Surg. 2020;49(1):187–99. doi: 10.1111/vsu.13356 31777975

[23] Pozzi A. TPLO, InternalBrace™ Ligament Augmentation: Theory and Rationale. In: Pozzi A, editor. Arthrex Vet Systems 2017.

[24] Dunn AL, Buffa EA, Marchevsky AM, Heller J, Moores AP, Farrell M. Inter- and intra-operator variability associated with extracapsular suture tensioning: an ex vivo study. Vet Comp Orthop Traumatol. 2012;25(6):472–7. doi: 10.3415/VCOT-11-12-0189 22829156

[25] Jevens DJ, DeCamp CE, Hauptman J, Braden TD, Richter M, Robinson R. Use of force-plate analysis of gait to compare two surgical techniques for treatment of cranial cruciate ligament rupture in dogs. Am J Vet Res. 1996;57(3):389–93. 8669774

[26] Lazar TP, Berry CR, deHaan JJ, Peck JN, Correa M. Long-term radiographic comparison of tibial plateau leveling osteotomy versus extracapsular stabilization for cranial cruciate ligament rupture in the dog. Vet Surg. 2005;34(2):133–41. doi: 10.1111/j.1532-950X.2005.00021.x 15860104

[27] Ledecky V, Hluchy M, Freilichman R, Hornak S, Knazovicky D. Clinical comparison and short-term radiographic evaluation of Tight Rope and Lateral Suture procedures for dogs after cranial cruciate ligament rupture. Veterinarni Medicina. 2014;59 (10):502–5.

[28] Brydges NM, Argyle DJ, Mosley JR, Duncan JC, Fleetwood-Walker S, Clements DN. Clinical assessments of increased sensory sensitivity in dogs with cranial cruciate ligament rupture. Vet J. 2012;193(2):545–50. doi: 10.1016/j.tvjl.2012.01.019 22386804

[29] Hunt JR, Goff M, Jenkins H, Harris J, Knowles TG, Lascelles BDX, et al. Electrophysiological characterisation of central sensitisation in canine spontaneous osteoarthritis. Pain. 2018;159(11):2318–30. doi: 10.1097/j.pain.0000000000001336 29994993 PMC6195806

[30] Knazovicky D, Helgeson ES, Case B, Gruen ME, Maixner W, Lascelles BD. Widespread somatosensory sensitivity in naturally occurring canine model of osteoarthritis. Pain. 2016;157(6):1325–32. doi: 10.1097/j.pain.0000000000000521 26901805 PMC4866583

[31] Harris LK, Whay HR, Murrell JC. An investigation of mechanical nociceptive thresholds in dogs with hind limb joint pain compared to healthy control dogs. Vet J. 2018;234:85–90. doi: 10.1016/j.tvjl.2017.12.012 29680400

[32] Murray MM, Kalish LA, Fleming BC, Team BT, Flutie B, Freiberger C, et al. Bridge-Enhanced Anterior Cruciate Ligament Repair: Two-Year Results of a First-in-Human Study. Orthop J Sports Med. 2019;7(3):2325967118824356. doi: 10.1177/2325967118824356 30923725 PMC6431773

[33] Murray MM, Fleming BC. Use of a bioactive scaffold to stimulate anterior cruciate ligament healing also minimizes posttraumatic osteoarthritis after surgery. Am J Sports Med. 2013;41(8):1762–70. doi: 10.1177/0363546513483446 23857883 PMC3735821

[34] DeCamp CE. Kinetic and kinematic gait analysis and the assessment of lameness in the dog. Vet Clin North Am Small Anim Pract. 1997;27(4):825–40. doi: 10.1016/s0195-5616(97)50082-9 9243783

